# Quantitative coronary computed tomography angiography for the detection of cardiac allograft vasculopathy

**DOI:** 10.1007/s00330-019-06653-3

**Published:** 2020-03-16

**Authors:** Borek Foldyna, Marcus Sandri, Christian Luecke, Jens Garbade, Robin Gohmann, Jochen Hahn, Julia Fischer, Matthias Gutberlet, Lukas Lehmkuhl

**Affiliations:** 1 Department of Interventional and Diagnostic Radiology, University of Leipzig – Heart Center, Struempellstrasse 39, 04289 Leipzig, Germany; 2 Cardiovascular Imaging Research Center, Massachusetts General Hospital – Harvard Medical School, Boston, MA USA; 3 Clinic for Radiology, Cardiovascular Center Bad Neustadt, Bad Neustadt, Germany; 4 Department of Cardiology, University of Leipzig – Heart Center, Leipzig, Germany; 5 University Department for Cardiac Surgery, Leipzig Heart Center, Leipzig, Germany

**Keywords:** Computed tomography angiography, Invasive coronary angiography, Cardiac allograft vasculopathy, Heart transplantation

## Abstract

**Objectives:**

To associate coronary wall volume and composition, derived from coronary computed tomography angiography (CTA), with cardiac allograft vasculopathy (CAV) detected on invasive coronary angiography (ICA) in heart-transplanted (HTX) patients.

**Methods:**

We included consecutive adults who received ICA and coronary CTA for evaluation of CAV ≥ 10 months after HTX. In all coronary segments, we assessed lumen and wall volumes and segmental length, calculated volume-length ratio (VLR) (volumes indexed by segmental length; mm^3^/mm), wall burden (WB) (wall/wall + lumen volumes; %), and assessed proportions of calcified, fibrotic, fibro-fatty, and low-attenuation tissue (%) in coronary wall*.* We rendered independent CTA measures associated with CAV by ICA, tested their discriminatory capacity, and assessed concordance between CTA and ICA.

**Results:**

Among 50 patients (84% men; 53.6 ± 11.9 years), we analyzed 632 coronary segments. Mean interval between HTX and CTA was 6.7 ± 4.7 years and between ICA and CTA 1 (0–1) day. Segmental VLR, WB, and proportion of fibrotic tissue were independently associated with CAV (OR = 1.06–1.27; *p* ≤ 0.002), reaching a high discriminatory capacity (combination of all three: AUC = 0.84; 95%CI, 0.75–0.90). Concordance between CTA and ICA was higher in advanced CAV (88%) compared with that in none (37%) and mild (19%) CAV. Discordance was primarily driven by a large number of segments with coronary wall changes on CTA but without luminal stenoses on ICA (177/591; 25%).

**Conclusion:**

CTA-derived coronary wall VLR, WB, and the proportion of fibrotic tissue are independent markers of CAV. Combination of these three parameters may aid the detection of early CAV not detected by ICA, the current standard of care.

**Key Points:**

*• Coronary CTA detects CAV in HTX patients.*

*• Coronary wall volume-length ratio, wall burden, and proportion of fibrotic tissue are independently associated with CAV.*

*• In contrast to ICA, coronary CTA may identify the early stages of CAV.*

**Electronic supplementary material:**

The online version of this article (10.1007/s00330-019-06653-3) contains supplementary material, which is available to authorized users.

## Introduction

Induced by immune and nonimmune triggers, cardiac allograft vasculopathy (CAV) reaches a high incidence of up to 40% in the first 5–8 years after heart transplantation (HTX) and represents the leading cause of graft failure and death among HTX patients [1]. According to recent guidelines, HTX patients should be frequently monitored by invasive coronary angiography (ICA) to detect CAV (class Ia recommendation [2, 3]). However, early CAV is characterized by vascular fibroproliferation and diffuse coronary wall changes without coronary stenosis, unlike advanced CAV, which presents with constrictive remodeling and coronary stenosis [4–7]. While ICA examines merely the coronary lumen, it focuses on stenosis and may miss early stages of CAV which occur as changes of the coronary wall. Supplemental invasive intravascular ultrasound (IVUS) can assess the coronary wall and increase sensitivity [3, 6, 8, 9]. However, IVUS is accompanied by an increased risk of severe complications (up to 4%), including coronary spasm, acute occlusions, embolism, arrhythmia, dissection, and acute thrombosis [8].

Coronary computed tomography angiography (CTA) represents a noninvasive alternative to ICA and IVUS [10–12] and has been successfully used in numerous large trials investigating coronary heart disease (CHD) [13–16] and in smaller studies of CAV [17–19]. Still, it remains unknown whether quantitative CTA measures are associated with CAV and how the diagnosis of CAV using these measures agrees with diagnosis made by ICA.

Thus, the primary aim of this study was to determine CTA-derived quantitative measures of coronary segments (i.e., volumes of the coronary lumen and wall as well as wall composition) in HTX patients and to investigate their association with CAV. Second, we aimed to determine the concordance between CTA and ICA and investigated quantitative CTA measures in all discordant cases.

## Materials and methods

### Study population

We included post-HTX adults who underwent routine ICA and coronary CTA for coronary wall imaging at our center for the exclusion (if CAV unknown) or for monitoring of CAV (if CAV known). The time interval between HTX and ICA was at least 10 months and no more than 7 days between ICA and CTA. Patients with acute allograft rejection, contraindications to contrast agents (e.g., severe renal dysfunction; blood creatinine > 1.5 mg/dl), or those who did not undergo ICA and CTA were not included. The local ethics committee approved this retrospective observational cohort study (IRB#442/18ek).

### CTA acquisition and coronary vessel segmentation

All CTA scans were performed on a second-generation 128-row dual-source CT scanner (Siemens Healthineers) using standard contrast-enhanced, ECG-gated/triggered protocols as described in detail in Supplemental Text S1. An expert reader (B.F.) with > 5 years of experience in coronary CTA analyzed all coronary segments (luminal diameter > 1.5 mm) following the 18-segment model (Society of Cardiac Computed Tomography; SCCT) [20] and using dedicated semi-automatic postprocessing software (QAngio, Research Edition, version 2.0.5; Medis Medical Imaging Systems). Left main, proximal right coronary artery, proximal left anterior descending, and proximal circumflex were considered proximal segments while the remaining as distal [21]. High reproducibility has been reported for this particular software package (wall burden intra-class correlation coefficient, 0.90 for intra- and 0.84 for inter-observer reliability [12, 19]). (Supplemental Figure S1 shows the individual standard analytic steps as described elsewhere [11, 12, 19, 22].)

### Quantitative coronary CTA analysis

First, we measured volume (mm^3^) (lumen and wall) and length (mm) of every coronary segment. Since long segments presented automatically with larger absolute volumes compared with short segments, we indexed all volumes by the length of the given segment. This step standardized the volumes and enabled comparison between segments with different lengths. All indexed volumes were treated as volume-length ratios (VLR; mm^3^/mm) in all analyses. Analogous to plaque burden calculation in CHD [12, 22], we calculated wall burden (WB), defined as a proportion of coronary wall relative to the size of the entire segment (i.e., wall volume/wall + lumen volume; %).

In a second step, we measured coronary wall composition using dynamic, tissue-specific attenuation thresholds as previously validated with IVUS [10–12, 23]. Hereby, four tissue types were assessed as absolute volumes (mm^3^): dense calcium, fibrotic, fibro-fatty, and low-attenuation tissue. To ensure comparability of segments of different sizes, we indexed all absolute values by the segmental wall volume and reported the composition as relative proportions (volume of individual proportion/wall volume; %).

Image quality was evaluated according to the 2014 SCCT guidelines for interpretation and reporting of coronary CTA using a 4-point Likert scale (1 = excellent; 2 = good; 3 = average; 4 = poor) [20]. Segments with poor image quality were excluded from further analysis.

### Invasive coronary angiography

All angiograms were acquired by experienced cardiologists using standard techniques according to the most recent guidelines and standard protocol in our clinic (Supplemental Text S2). An expert reader (M.S.) with > 6 years of ICA experience interpreted all angiograms, was blinded to all CTA findings, and used the SCCT 18-segment model [20] to ensure comparability with CTA. All coronary lesions were classified visually according to stenosis grade and type as recommended by the American College of Cardiology/American Heart Association (ACC/AHA) Task Force guidelines (grade 0, 0–24%; 1, 25–49%; 2, 50–74%; 3, 75–90% and > 90%; 4, 100% and type A, < 10 mm length, concentric; B, 10–20 mm length, eccentric; and C, > 20 mm length, excessive tortuosity) [24]. We classified CAV-positive segments as follows: *no CAV*, no stenosis or stenosis 1–24%; *mild CAV*, stenosis grade 25–49%, type A or B; *≥ moderate CAV*, stenosis ≥ 50% and type A–C.

### Statistical analysis

Continuous variables were expressed as mean ± standard deviation (SD) or median (interquartile range; IQR), while categorical variables were listed as frequencies and percentages. To increase readability, we tabulated all values as mean ± SD and restricted the use of the median (IQR) to highly skewed or ordinal-scaled data. Differences of CTA measures between CAV-positive vs. CAV-negative patients were tested with the Wilcoxon rank-sum or Fisher’s exact test, as appropriate. Associations of individual CTA measures with CAV by ICA were tested with univariate logistic regression. Subsequently, we rendered independent CTA variables using multivariate regression, including all variables which were associated with CAV in the univariate analyses (*p* < 0.05). In a supplemental analysis, we provided univariate and multivariate results adjusted for segment location (proximal vs. distal).

After building a multivariate logistic regression model, which included all CTA variables independently associated with CAV, we calculated relative probability of having CAV in each coronary segment (continuous measure with range, 0–1; equation provided in Supplemental Text S3). Using these probabilities, we computed areas under receiver operator curve (AUC/ROC) as measures of discriminatory capacity to detect CAV. Differences between AUC curves were tested with the likelihood-ratio test for nested models. In an explorative analysis, we estimated a threshold for the CTA-derived probability using the Youden index [25] (equal weight on sensitivity and specificity) including sensitivity and specificity for the given threshold.

Finally, we categorized the CTA-based probability into tertiles (i.e., low, intermediate, and high) and tabulated these against the CAV grades on ICA (none, mild, and ≥ moderate). We assessed the concordance between CTA and ICA and identified groups of segments with the highest concordance and discordance. Level of significance was defined as two-sided *p* < 0.05, and Stata 15.0 was used for all statistical analyses.

## Results

### Study population and evaluable coronary segments

Among 42 (84%) men and 8 (16%) women (mean age of 53.6 ± 11.9 years), who were included in the analytic cohort, CAV prevalence was 38% (19/50) on ICA. The mean interval between HTX and CTA was 6.7 ± 4.7 years, while the median interval between ICA and CTA was 1 (0–1) day. Dilative cardiomyopathy (58.0%) was the most frequent indication for HTX, and the most common comorbidities were renal dysfunction (44%), dyslipidemia (26%), and diabetes mellitus (22%). Detailed demographics and medical treatment information are provided in Table 1 and Supplemental Table S1, respectively.

**Table 1:** Patient characteristics

Demographics	All (*n* = 50)
Age, years	53.4 ± 12.0
Male, *n* (%)	42 (84)
BMI, kg/m^2^	27.0 ± 3.9
Primary indication for HTX, *n* (%)	
Dilatative cardiomyopathy	29 (58)
Ischemic heart disease	16 (32)
Other	5 (10)
Cardiovascular risk factors, *n* (%)	
Diabetes	11 (22)
Art. hypertension	6 (12)
Hyperlipidemia	13 (26)
Atrial fibrillation	4 (8)
Peripheral artery disease	2 (4)
Renal disorder	22 (44)
Current smoker	2 (4)

*BMI* body mass index

Despite relatively high heart rate (mean 74.1 ± 8.5 bpm), the median image quality was rated as good (IQR, excellent–good); (correlation of heart rate with image quality, *r* = 0.28; *p* = 0.05). Ultimately, 56 segments had poor image quality and were excluded from the analysis. Median dose length product and effective dose were 413.5 (225.0–678.0) mGy cm and 5.8 (3.2–9.5) mSv, respectively.

Overall, 692 coronary segments were evaluable on CTA, and among these, 632/692 (91.4%) segments had a corresponding partner on ICA. Hence, the final analytic sample consisted of 632 coronary segments, of which 190/632 (30.1%) and 442/632 (69.9%) segments were classified as proximal and distal, respectively.

### Quantitative coronary CTA analysis

On CTA, the mean wall and lumen VLRs were 7.7 ± 3.2 and 5.8 ± 4.7 mm^3^/mm, respectively, while the mean WB was 59.1 ± 8.6% (Table 2). WB was higher in distal coronary segments (62% vs. 56%; *p* < 0.001), while coronary lumen and wall VLRs were twofold lower compared with the proximal segments (lumen, 4.4 ± 3.1 vs. 9.5 ± 6.0 mm^3^/mm, and wall, 6.7 ± 2.5 vs. 10.0 ± 3.3 mm^3^/mm; *p* < 0.001 for both; Supplemental Table S2).

**Table 2:** Quantitative CTA measures of coronary segments stratified by CAV status diagnosed on ICA

CTA measures	All segments (*n* = 632)	CAV-negative on ICA (*n* = 591)	CAV-positive on ICA (*n* = 41)	*p*
Volume-length ratio, mm^3^/mm				
Lumen	5.8 ± 4.0	5.8 ± 4.7	5.9 ± 5.0	0.864
Wall	7.7 ± 3.2	7.5 ± 2.9	10.6 ± 4.5	< 0.001
Wall burden, %	59.1 ± 8.6	59.5 ± 8.4	66.3 ± 10.0	< 0.001
Composition, %				
Dense calcium	1.0 ± 2.8	0.8 ± 2.3	4.2 ± 6.1	< 0.001
Fibrotic tissue	44.7 ± 11.2	44.4 ± 11.1	48.7 ± 11.4	0.033
Fibro-fatty tissue	18.6 ± 7.8	18.7 ± 7.8	17.4 ± 7.4	0.204
Low-attenuation tissue	8.5 ± 6.6	8.6 ± 6.7	7.1 ± 6.1	0.163

*CAV* cardiac allograft vasculopathy; *CTA* computed tomography angiography; *ICA* invasive coronary angiography

Regarding composition, coronary walls consisted of 44.7% fibrotic, 18.6% fibro-fatty, 8.5% low-attenuation, and 1.0% calcified tissue (Table 2), while the remaining 27.2% were outside of the tissue-specific thresholds (e.g., media border). Here is to be noted that the proportion of fibrotic tissue was significantly higher, while proportions of calcified, fibro-fatty, and low-attenuation tissue were significantly lower in the distal compared with that in proximal coronary segments (*p* < 0.001 for all; Supplemental Table S2).

### Invasive coronary angiography

On ICA, 19/50 (38%) patients and 41/632 (6.5%) coronary segments were rated as CAV-positive. Among patients with at least one coronary lesion, median 1 (1–3) segments were classified as CAV-positive on ICA. Single-, double-, and triple-vessel diseases were present in 12/19 (63%), 4/19 (21%), and 3/19 (16%) patients, respectively. Median stenosis grade was 2 (2–2) (50–75% luminal stenosis), while the most frequent lesion type was B (25/49; 51%) followed by C (18/49; 37%) and A (6/49; 12%).

### Quantitative coronary CTA measures in segments with and without CAV on ICA

Coronary wall VLR was significantly larger in the 41 CAV-positive segments compared with those without CAV (10.6 ± 4.5 vs. 7.5 ± 2.9 mm^3^/mm; *p* < 0.001). On the other hand, coronary lumen VLR did not differ significantly between CAV-positive and CAV-negative segments (*p* = 0.864). Accordingly, WB was higher in segments with CAV compared with those without CAV (66.3 ± 10.0 vs. 59.5 ± 8.4%; *p* < 0.001).

Regarding the wall composition, CAV-positive coronary segments had more fibrotic tissue (48.7 ± 11.4 vs. 44.4 ± 11.1%; *p* = 0.033) and dense calcium (4.2 ± 6.1 vs. 0.8 ± 2.3%; *p* < 0.001) compared with CAV-negative segments. Proportions of fibro-fatty and low-attenuation tissue did not differ between CAV-positive and CAV-negative segments (*p* = 0.204 and 0.163, respectively) (Table 2).

In the univariate logistic regression, coronary wall VLR, WB, and calcified and fibrotic tissue were associated with CAV (OR = 1.04–1.28; *p* ≤ 0.019 (ORs per mm^3^/mm or % increase as appropriate) (Table 3). In the multivariate analysis, coronary wall VLR, WB, and fibrotic tissue remained independently associated with CAV (OR = 1.27; *p* < 0.001, OR = 1.09; *p* < 0.001, and OR = 1.06; *p* = 0.002, respectively) (Table 3). All three CTA measures remained significantly associated with CAV after further adjustment for segment location (*p* ≤ 0.002 for all; Supplemental Table S3).

**Table 3:** Association of CTA parameters with presence of CAV on ICA

*n* = 632 segments; CAV-positive *n* = 41	Univariate			Multivariate*		
	OR	95% CI	*p*	OR	95% CI	*p*
Volume-length ratio (per mm^3^/mm)						
Lumen	1.01	0.94–1.07	0.876	–	–	–
Wall	1.28	1.17–1.40	< 0.001	1.27	1.14–1.42	< 0.001
Wall burden (per %)	1.13	1.08–1.19	< 0.001	1.09	1.05–1.14	< 0.001
Composition, relative (per %)						
Dense calcium	1.20	1.12–1.28	< 0.001	1.08	1.00–1.18	0.051
Fibrotic tissue	1.04	1.01–1.07	0.019	1.06	1.02–1.10	0.002
Fibro-fatty tissue	0.98	0.94–1.02	0.313	–	–	–
Low-attenuation tissue	0.96	0.91–1.01	0.154	–	–	–

Coronary wall VLR, wall burden and the proportion of dense calcium and fibrotic tissue were associated with CAV. Notably, the association of fibrotic tissue with CAV increased in the multivariate analysis, emphasizing its independence of the other factors. *Includes all variables *p* < 0.05 in the univariate analysis. *CAV*, cardiac allograft vasculopathy; *CI*, confidence interval; *OR*, odds ratio

**Fig. 1:**
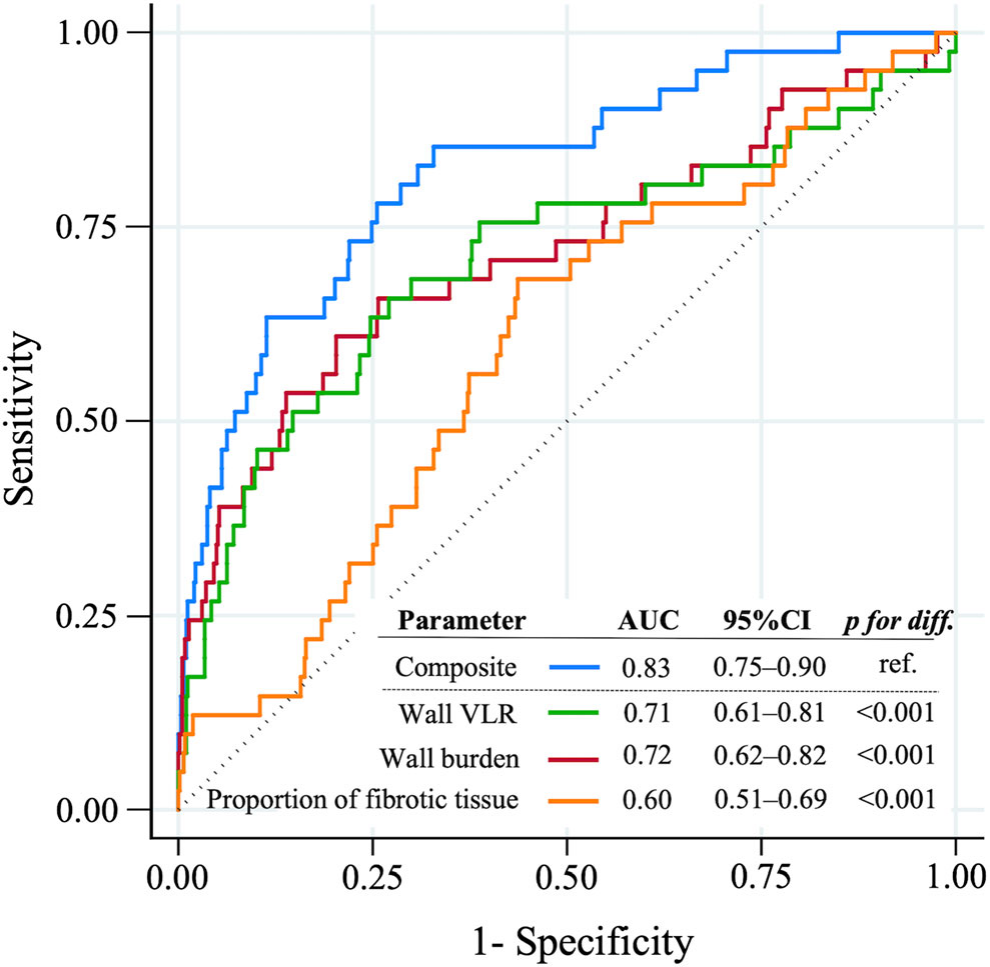
Discriminatory capacity of quantitative CTA for the detection of CAV. Receiver operator characteristics curves for individual quantitative CTA measures (coronary wall volume-length ratio (VLR; mm^3^/mm), wall burden (%), and fibrotic tissue proportion (%)) and the composite of all three parameters (i.e., logistic regression–based probability). The composite reached the highest discriminatory capacity. *p* values indicate significantly larger AUC (0.83) compared with the AUCs of the individual CTA measures. VLR, volume-length ratio

Mean CTA-derived probability to have CAV was 6 ± 11% (range 0–97%) (derived from logistic model nesting coronary wall VLR, WB, and fibrotic tissue; Supplemental Text S3 provides the corresponding formula for calculation of individual combined probability). The probability reached a high discriminatory capacity for CAV (AUC = 0.83; 95%CI, 0.75–0.90) (Fig. 1). Even though the quantitative CTA did not focus on accessing luminal stenosis and, therefore, was not directly comparable with ICA, in a direct exploratory comparison of CTA vs. ICA, the sensitivity and specificity were relatively high (78% and 75%, respectively, by using a threshold of 5% calculated by the Youden index).

### Concordance CTA vs. ICA

**Fig. 2:**
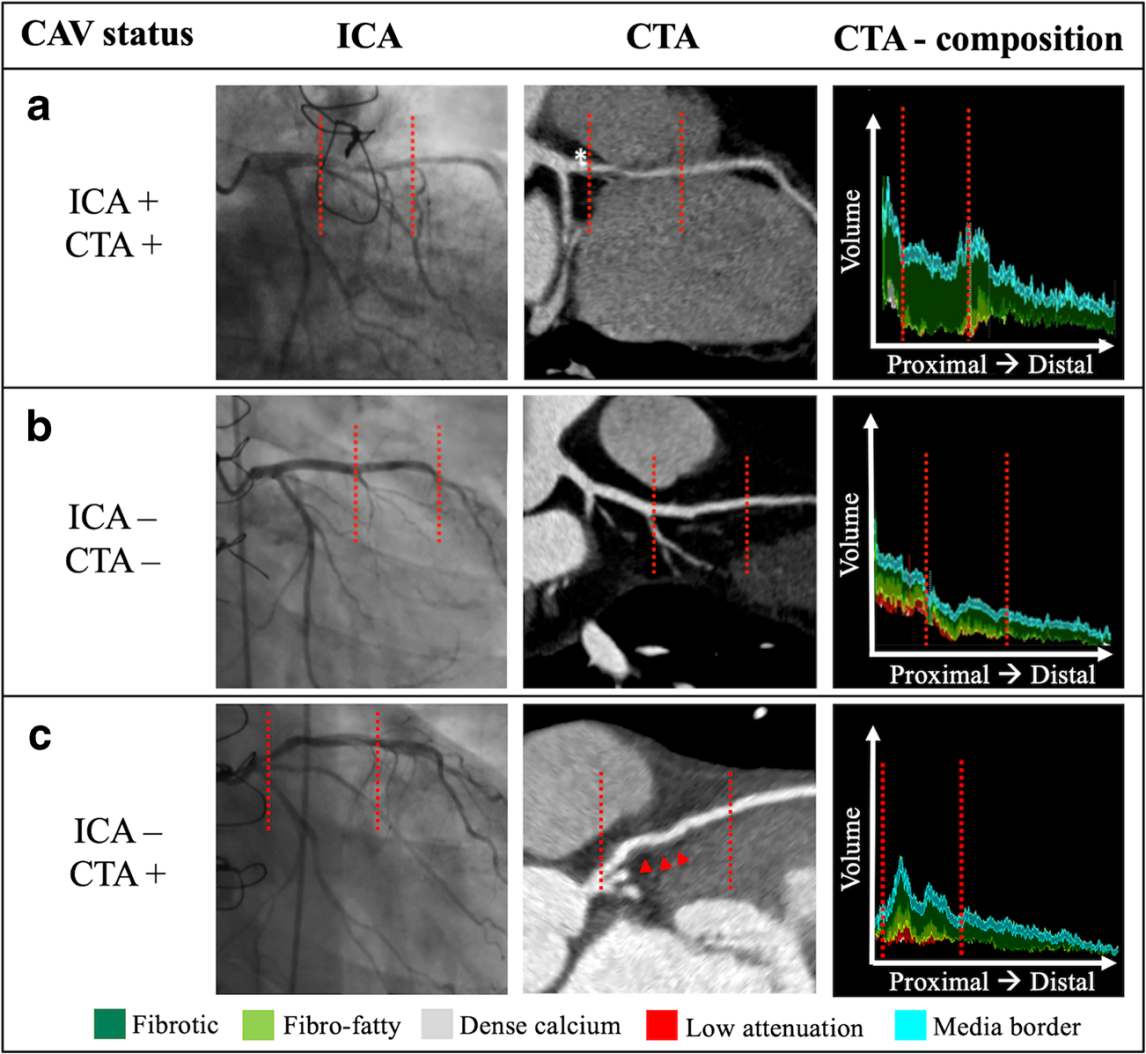
Imaging examples stratified by CAV status. Left coronary arteries of three patients with (**a**) CAV on ICA and CTA, corresponding luminal stenoses on ICA and wall thickening on CTA; (**b**) patient without signs of CAV, with normal coronary lumen on ICA and thin coronary wall on CTA; and (**c**) patient with normal coronary lumen on ICA (also no stenosis on CTA), but wall thickening (red arrowheads) on CTA. Red dotted lines mark the reference areas of interest. CAV, cardiac allograft vasculopathy; CTA, computed tomography angiography; ICA, invasive coronary angiography

Overall, there was a moderate concordance of 37% (233/632) between the coronary CTA and ICA (Table 4). As expected, the concordance was substantially higher in those with advanced disease (22/25 (88%)) compared with those with none or mild disease on ICA (208/591 (37%) and 3/16 (19%), respectively). Focusing on the extremes, namely the difference of two categories, coronary CTA classified 177/632 (28%) segments as a high probability for having CAV without a corresponding correlate on ICA. Not surprisingly, the discordant 177 segments had higher coronary wall VLR, WB, and more fibrotic tissue (*p* < 0.001 for all) compared with segments with low and intermediate probability and no CAV on ICA. Figure 2c provides an example of a discordant case. Moreover, the discordant segments were more frequently distal segments (112/177 (63%) vs. 65/177 (37%); *p* = 0.012). On the other hand, only 1/632 (0.2%) segment was classified as a low probability by CTA but presented with ≥moderate CAV on ICA.

**Table 4:**
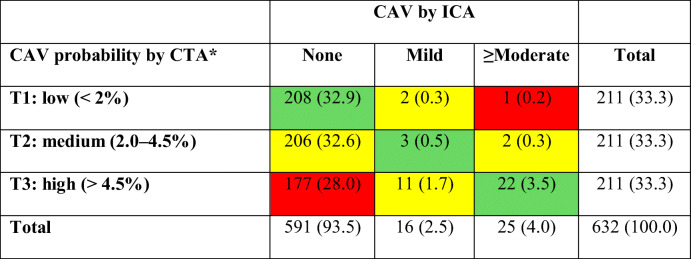
CTA vs. ICA for the detection of CAV

The discordance between CTA and ICA was driven by segments which have shown large coronary wall changes on CTA but were not classified as CAV-positive by the ICA (*n* = 177; red lower left field). *Based on the logistic regression including coronary wall volume-length ratio, wall burden, and proportion of fibrotic tissue. *CAV* = cardiac allograft vasculopathy; *CTA* = computed tomography angiography; *ICA* = invasive coronary angiography

## Discussion

In this study, we investigated the association of quantitative coronary CTA with CAV and explored the concordance between CTA and ICA. This study had two major findings: (1) CTA-derived coronary wall volume-length ratio, wall burden, and proportion of fibrotic tissue were independently associated with CAV, and (2) concordance between CTA and ICA was high in advanced CAV, while discordance was driven predominantly by distal coronary segments with changes of coronary wall on CTA but without CAV on ICA. Our study identified the CTA-derived coronary wall VLR, WB, and proportion of fibrotic tissue as independent measures of CAV; corroborated the high performance of CTA to detect advanced CAV; and revealed a potential advantage of quantitative CTA in early stages of CAV.

Due to rapid technical progress, coronary CTA has become an alternative to ICA for the exclusion of CHD in patients with chest pain [13–15, 26, 27] and has received Ib recommendation in the most recent 2019 European Society of Cardiology guidelines for the diagnosis and management of chronic coronary syndromes [28]. However, coronary CTA has not become a routine procedure to detect CAV in HTX patients yet (class IIb recommendation [3]). In HTX patients, the primary concern is image quality. The limiting factor, influencing the image quality, is the frequently observed high heart rate and a limited response of HTX patients to heart rate modulation (e.g., beta-blockers).

While in older scanners image quality depended strongly on the heart rate [29–31], recent studies have shown that contemporary CTA technology reaches diagnostic image quality in HTX patients with heart rates > 70 bpm at reasonable radiation exposure (4.3–6.6 mSv) [18, 19, 32, 33]. Our study endorses these results by revealing good image quality at a mean heart rate of 74 bpm and median effective radiation dose of 5.8 mSv. One could argue that the effective dose in CTA is higher than the reported average median effective dose of diagnostic ICA (3.3 mSv) [34]. Nevertheless, several recent studies using the most recent CTA technology have reported excellent images at effective dose of 0.3–2.9 mSv despite heart rates above 70 bpm or heart rhythm irregularities [35–37]. Therefore, further advances in temporal resolution and introduction of novel protocols, combining low kVp and iterative reconstruction algorithms, will likely lead to a further decline of radiation necessary for diagnostic image quality in HTX patients.

Considering the technical improvement of coronary CTA, it is not surprising that qualitative detection of obstructive coronary stenoses (≥ 50%) on CTA agrees well with ICA not only in patients with CHD but also in HTX patients (negative predictive value, 98–100%) [17, 27, 33, 38, 39]. Quantitative coronary wall analysis has become a supplementary tool to qualitative CTA by providing additional information about coronary wall volume and composition. In patients with CHD, quantitative CTA has revealed a good agreement with IVUS [11, 12] and has become a standard to monitor changes of atherosclerotic plaques in numerous studies using serial CTA [40–43]. Nevertheless, there is only limited evidence about the utilization of quantitative CTA in HTX patients. For instance, Karolyi et al used serial CTA to show that quantitative assessment of coronary walls is feasible to measure coronary wall changes over time and that progression of coronary wall volume is driven by noncalcified components [19]. Our study underlines the feasibility and enhances these results by identifying coronary wall VLR, WB, and proportion of fibrotic tissue as independent indicators of CAV.

Regarding pathophysiology of CAV, our results confirmed a series of histopathological studies which have shown that CAV is primarily driven by fibroproliferation [4–7], reflected in a higher proportion of fibrotic tissue in CAV-positive segments. Moreover, CAV has been reported to progress typically in a biphasic manner. Namely, CAV progression starts with an initial phase, including early intimal thickening with an expansion of the external elastic membrane and relative preservation of the lumen. The initial phase is then followed by an advanced phase with constrictive remodeling and luminal stenosis [5, 7]. While ICA captures merely the coronary lumen, it may miss the early phases of CAV. The fact that CTA enables measurements of coronary walls regardless of luminal stenosis may explain why our sensitivity and specificity of quantitative CTA (78% and 75%, respectively) were lower than the usually reported values for qualitative CTA (assessment of significant stenosis (≥ 50%); 94% and 92%, respectively) [33].

The reason becomes even more apparent if investigating the concordance between quantitative CTA and ICA. We found a high concordance (88%) between both methods in advanced CAV, which confirms that lesions with coronary wall changes and lumen narrowing are detectable by both methods accurately. These results are in accord with a meta-analysis by Wever-Pinzon et al, who investigated the accuracy of qualitative CTA to detect CAV in comparison with ICA [33]. However, the discordance was driven by segments which were classified as CAV-positive by CTA and CAV-negative by ICA (i.e., segments with coronary wall changes but without a luminal stenosis; example, Fig. 2c). In fact, quantitative CTA has shown significant coronary wall changes in 28% of segments rated as normal on ICA (Tab. 4). Vice versa, CTA rated only one (0.2%) coronary segment as a low probability of having CAV while the ICA reported advanced CAV. These findings are striking and suggest a higher sensitivity and a higher negative predictive value of quantitative CTA for the detection of early CAV compared with ICA. Future prospective studies with less mechanistic and more prognostic endpoints (e.g., survival) are needed to confirm this hypothesis.

Moreover, the discordance between CTA and ICA was the highest in distal coronary segments, those not always accessible by IVUS, underlining the importance to evaluate the entire coronary tree, representing a clear advantage of CTA compared with IVUS. Indexing of segmental volumes by segmental length (i.e., VLR) and treating coronary wall components as proportions standardized the values and permitted comparison of segments at different locations. In a supplemental analysis, we showed that segmental VLR, WB, and proportion of fibrotic tissue are all independently associated with CAV even after adjustment for segment location. The independence of segment location further emphasizes the robustness and generalizability of the quantitative CTA measures.

In general, coronary walls and lumen were smaller, while WB and proportion of fibrotic tissue were higher in the distal coronary segments compared with those in the proximal segments. In other words, in the course of the vessel, the lumen became smaller while the wall did not get necessarily thinner. This observation should get further attention in future studies to investigate whether an increasing WB towards distal coronary segments reflects normal anatomy or also a sign of CAV.

Potential clinical implications of our results may include earlier detection of CAV leading to a sooner initiation or more aggressive regimen of medical therapy, intensification of follow-up visits, and more intensive management of risk factors. Notably, in our cohort, > 80% of patients were on NSAR and statins, and around 60% received tacrolimus/everolimus, which seem to be the most effective treatment/prevention strategy for CAV. While the long-term efficacy of these last-named medications remains unclear [44], quantitative CTA may represent a novel tool to monitor not only the progression of CAV but also the effects of immunosuppressive drugs. In fact, quantitative coronary CTA has been successfully utilized for monitoring of coronary plaques in patients receiving statins [40, 45, 46] in contrast to earlier studies, such as the SATURN trial [47], which traditionally relayed on IVUS.

We acknowledge several limitations of this retrospective single-center observational cohort study. First, we included only HTX patients and did not compare the CTA results with general population. Thus, future studies, preferably in individuals without known CHD, should define normal values of VLR, WB, and fibrotic tissue. We did not have information about the cardiovascular risk profile of our heart donors, and preexisting CHD may have influenced our measurements (e.g., calcifications). Serial approaches, which would include a baseline CTA exam and would focus on changes of the qualitative CTA measures during follow-up, may provide more insights into the pathogenesis of CAV. Moreover, the sample size in our study was relatively small. Future larger studies should include the evaluation of clinical outcomes and investigate the incremental value of quantitative over qualitative CTA.

## Conclusion

Coronary CTA-derived wall VLR, WB, and proportion of fibrotic tissue were independently associated with CAV on ICA. While the concordance between CTA and ICA was high in advanced CAV, the discordance was mainly driven by distal coronary segments showing changes of coronary walls on CTA but without corresponding stenosis on ICA. These segments may represent those with non-stenotic coronary wall changes, related to early stages of CAV. Thus, quantitative CTA, in particular the combination of VLR, WB, and proportion of fibrotic tissue, may aid the detection of early CAV, guide medical treatment, and ultimately improve patients’ outcomes.

## Electronic supplementary material

ESM 1 (DOCX 212 kb)

## Abbreviations

AUC: Area under the curve
CAV: Coronary allograft vasculopathy
CHD: Coronary heart disease
CTA: Computed tomography angiography
HTX: Heart transplantation
HU: Hounsfield units
ICA: Invasive coronary angiography
IQR: Interquartile range
IVUS: Intravascular ultrasound
ROC: Receiver operating characteristic
SCCT: Society of Cardiovascular Computed Tomography
SD: Standard deviation
VLR: Volume-length ratio
WB: Wall burden
